# The microbial community shifts of subgingival plaque in patients with generalized aggressive periodontitis following non-surgical periodontal therapy: a pilot study

**DOI:** 10.18632/oncotarget.12532

**Published:** 2016-10-08

**Authors:** Jing Han, Peng Wang, Shaohua Ge

**Affiliations:** ^1^ Shandong Provincial Key Laboratory of Oral Tissue Regeneration, School of Stomatology, Shandong University, Jinan, China; ^2^ Department of Periodontology, School of Stomatology, Shandong University, Jinan, China

**Keywords:** generalized aggressive periodontitis, metagenome, subgingival plaque, non-surgical treatment, bacterial community shifts

## Abstract

The object of this study is to characterize the bacterial community of subgingival plaque of two subjects with generalized aggressive periodontitis (GAgP) pre- and post-treatment. We picked two patients with GAgP and used high-throughput 16S rDNA sequencing. V4 hypervariable region was picked for PCR amplification of subgingival samples. Then, the PCR products were sequenced through Illumina MiSeq platform. One month after therapy, both the clinical features and periodontal parameters improved obviously. Moreover, the composition and structure of subgingival bacterial community changed after initial periodontal therapy. Also, the composition of the subgingival microbiota was highly individualized among different patients. *Bacteroidetes*, *Spirochaetes* and *Fusobacteria* were related to pathogenicity of GAgP while *Actinobacteria* and *Proteobacteria* seemed associated with clinical symptoms resolution. In this study, we found the subgingival bacterial community was high in species richness but dominated by a few species or phylotypes, with significant shifts of microbiota that occurred after treatment. This study demonstrated the shift of the subgingival bacterial community before and after treatment by high-throughput 16S rDNA sequencing, and provided a concise method for analysis of microbial community for periodontal diseases.

## INTRODUCTION

The oral cavity contains a large number of ecological sites that provide surfaces for microbial colonization. Studies revealed that the predominant bacteria were normal flora, which were non-pathogenic and helped prevent colonization by exogenous organism, and were considered to be commensal in the oral cavity [1, 2]. However, some of the bacteria are associated with oral diseases, such as dental caries and periodontal diseases. By culture method, approximately 200 bacterial species have been found in the oral cavity. Using a metagenomic approach first proposed by Kroes et al [3] and Paster et al [4], over 1000 phylotypes have been detected in the oral samples [5], which raised the possibility that uncultivated and as-yet-uncharacterized species might also participate in the etiology of oral diseases [6]. It is now well recognized that periodontitis is a kind of polymicrobial infection. However, only about half of the subgingival bacterial species or phylotypes are cultivable, which presents an obstacle to fully understand the causal relationship between subgingival bacteria and periodontitis [7]. Most of our understanding of subgingival bacteria comes from cultivable bacteria, and the role of many non-cultivable bacteria remains largely unknown. Furthermore, whether the microbial community shifts before and after periodontal therapy remains unclear. Metagenomic study involves the identification, sequencing, and functional and transcriptome analysis of environmental samples. Using metagenomic methods, the members of the microbial community are typically determined by tracking phylogenetic markers such as the 16S rDNA.

GAgP represents a specific type of periodontitis with clearly identifiable clinical and laboratory findings that are different from other forms of periodontitis. GAgP is characterized by the relatively young ages of the affected individual (usually 30 years old or older), and a generalized loss of attachment and alveolar bone except in incisors, first molars and other permanent teeth (besides at least three permanent teeth) [8]. Actually, GAgP usually affected most teeth of oral cavity. It has been revealed that the predominant bacteria of GAgP include *Actinobacillus actinomycetemcomitans (Aa), Porphyromonas gingivalis (Pg), Tannerella forsythensis (Tf), Treponema denticola (Td), Campylobacter rectus (Cr), Prevotella intermedia (Pi) and Prevotella nigrescens (Pn)* [9,10]. However, the microbial community shifts pre- and post-treatment of GAgP is still unclear.

Therefore, our present study aimed to elucidate explorative and descriptive shifts in bacterial communities by next generation sequencing of subgingival plaque samples from two GAgP patients before and after mechanical debridement. The hypervariable region V4 of the 16S rDNA gene was targeted to explore richness and diversity of subgingival plaque samples [11].

## RESULTS

In this study, we analyzed subgingival plaque samples of two patients with GAgP for shifts in the microbial community in response to mechanical debridement. Demographic data, baseline and post intervention clinical parameters probing depth (PD) and bleeding on probing (BOP) were listed in Table S1. All clinical parameters improved after treatment. The intraoral photos and panoramic radiographs of two patients before and after treatment were shown in Figure S2 and Figure S3.

**Table 1:** Comparison of the estimated operational taxonomic unit (OTU) richness, diversity index and Simpson index of 16S rDNA gene libraries for clustering at 97% identity as obtained from the pyrosequencing analysis

Samples	Sequences number	Shannon	Chao1	Simpson index
G1	1190.0	6.00	212.3	0.75
G2	1190.0	6.23	220.0	0.75
G3	1190.0	6.25	219.5	0.76
G4	1190.0	6.77	352.2	0.78
GT1	1190.0	5.63	323.5	0.68
GT2	1190.0	6.14	349.8	0.77
GT3	1190.0	5.81	334.6	0.75
GT4	1190.0	6.09	370.5	0.75
Z1	1190.0	5.18	184.0	0.78
Z2	1190.0	4.86	171.7	0.78
Z3	1190.0	5.74	190.2	0.73
Z4	1190.0	4.98	170.7	0.75
ZT1	1190.0	3.99	154.6	0.69
ZT2	1190.0	3.32	117.7	0.66
ZT3	1190.0	2.42	147.3	0.42
ZT4	1190.0	2.17	204.1	0.30

G and Z represented the samples from the patient G and Z before treatment; GT and ZT represented the samples from the patient G and Z after treatment.

### Filtration and quality evaluation of original data

**Figure 1:**
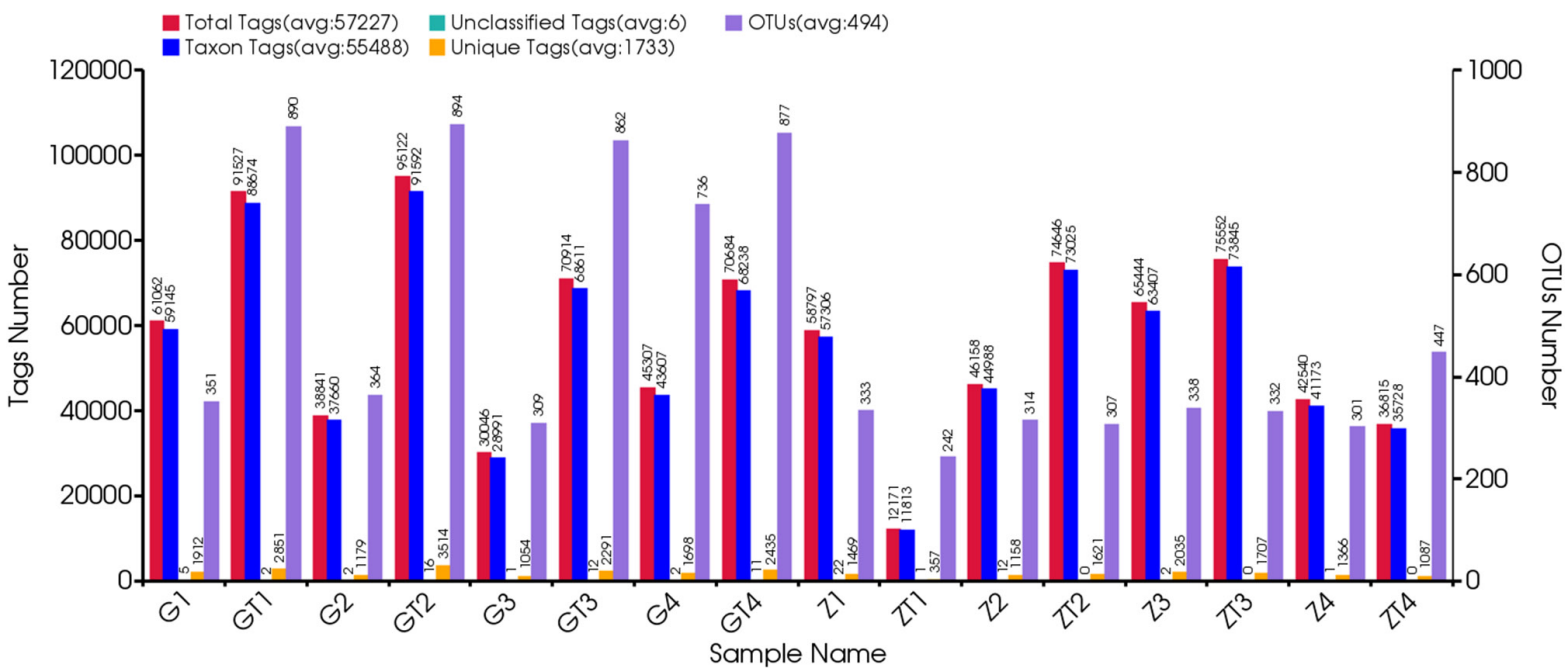
Statistical information of original data The first vertical axis (Tags numbers):Total Tags means the number of merged tags after filtering; Taxon Tags means the number of tags which were used for establishing OTU table and obtained taxonomic information; Unclassified Tags means the number of tags which were used for establishing OTU table but did not obtain taxonomic information; Unique Tags means the number of tags whose frequency was 1 and can't be assigned to any OTUs. The second vertical axis (OTUs Number) represents the final OTU number after taxonomic analysis.

A total of 952,272 V4 16s rDNA paired-end reads were obtained from the 16 samples (the minimum and maximum numbers of reads from the 16 samples were 12,564 and 100,089, respectively) (Figure 1). All of the 16 samples were used in this analysis. The raw reads were filtrated by QIIME quality filters. After filtrating, 915,626 sequence reads were left. The average length of the filtered sequence reads was 253 bp (Table S2).

### Operational taxonomic unit-based analysis

**Figure 2:**
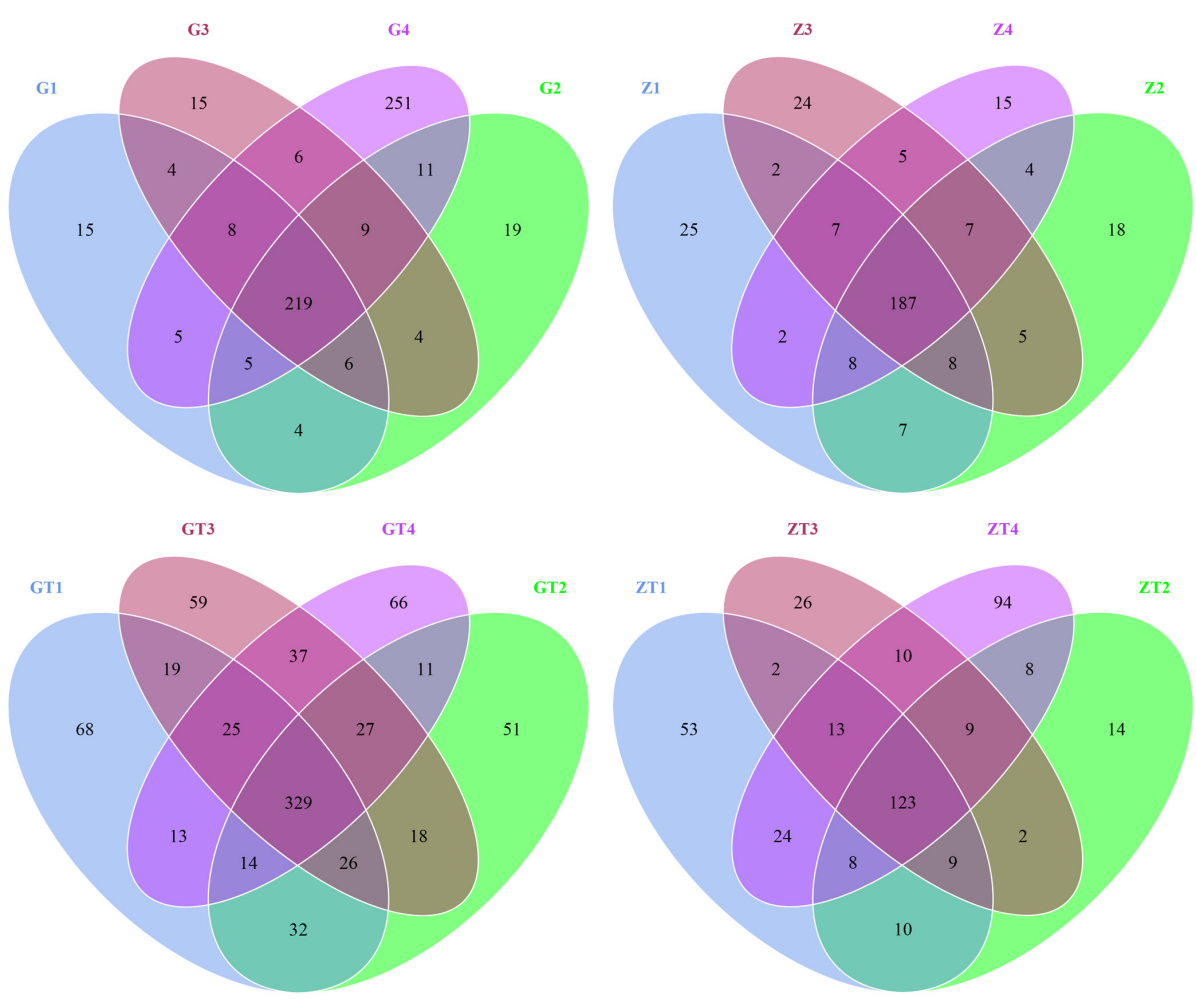
Scalar-Venn representation of shared genera among microbiomes associated with 4 sites in two patients before and after treatment G1-4 and Z1-4 meaned the samples from patient G and patient Z before treatment respectively. GT1-4and ZT1-4 meaned the samples from the patient G and patient Z after treatment (represent as GT and ZT) respectively. The overlap section represented the shared OTUs between different samples.

The taxon abundance of all 16 samples was generated into 34 phyla, 82 classes, 127 orders, 156 families and 187 genera. First, all operational taxonomic units (OTUs) picked from the 16 samples were normalized by homogenization procedure. Then, they were contrasted and hierarchical clustered intragroup mainly by using RDP classifier. Finally, we got Vene diagrams on this basis (Figure 2). The OTUs in each sample and the number of sequences in each OTU were counted to obtain the taxonomic information of the OTU. The percentage of shared OTUs among groups after treatment decreased. In contrast, the percentages of particular OTUs increased (Table S3). We supposed that prevalent microorganisms of GAgP suppressed the growth of other microorganisms which led to an increasing percentage of pathogenic microorganisms in microflora before treatment. After periodontal debridement, the growth of pathogenic microorganisms was suppressed, and the proportion of the pathogenic microorganisms decreased accordingly.

### Alpha diversity analysis

**Figure 3:**
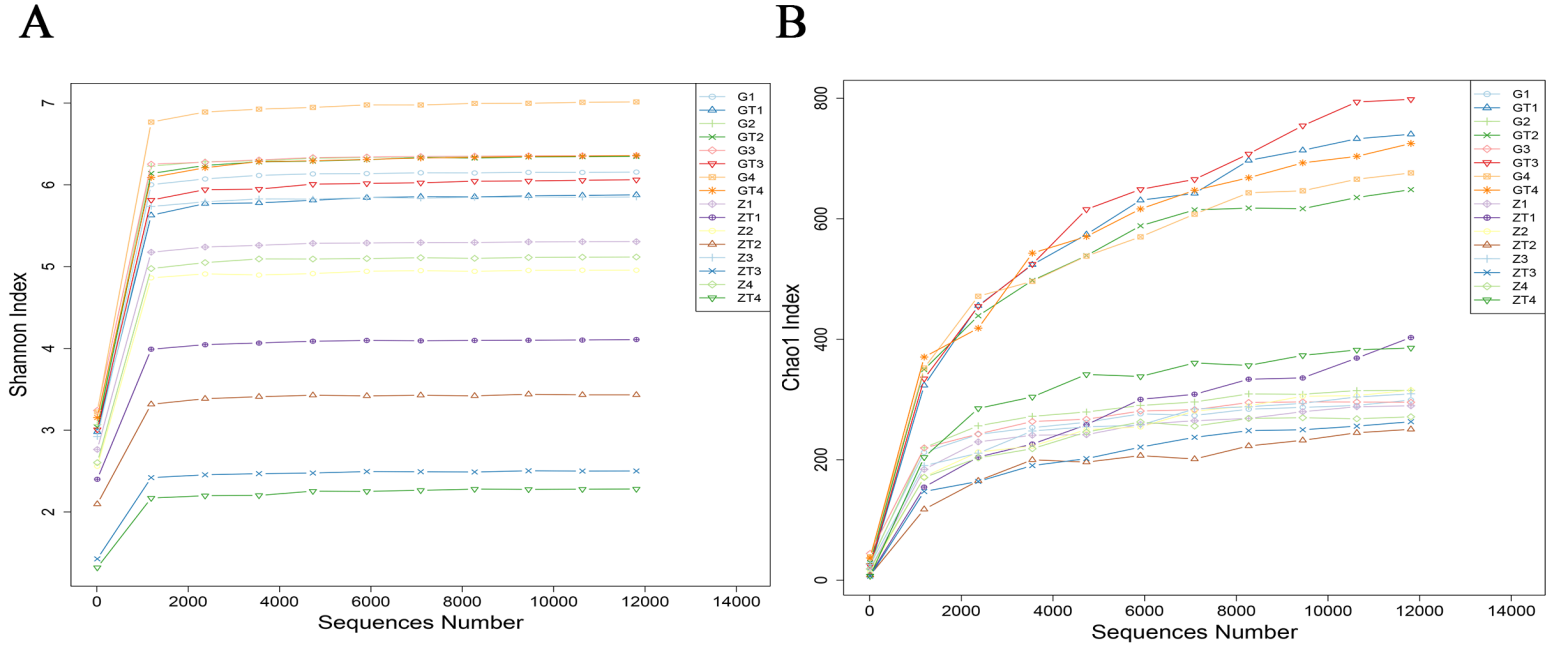
The rarefraction curves of Shannon index **A**. and Chao1 index **B**. The horizontal axis represented sequence number, the vertical axis represented the shannon index in **A**. and chao1 index in **B**.

Alpha diversity estimates the diversity in a specific area or ecosystem in terms of species richness which is estimated by rarefaction curves. The shape of the rarefaction curves indicates new phylotypes which will be expected with additional sequencing. The Shannon and Chao1 index curves of all samples reached plateaus with the current sequencing (Figure 3A, 3B), suggesting that most richness and diversity had already been captured. The subgingival plaques richness index was calculated as shown in Table 1. The Shannon index of diversity reflects both diversity and community evenness, and the Chao1 index is an estimator of phylotype richness. Lower Shannon index after treatment indicated that the treatment reduced bacterial diversity within the subgingival plaque. Higher Chao1 index after treatment indicated that treatment increased the bacterial richness of the subgingival plaque. These suggested that the group after treatment had a higher level of biodiversity and unevenness estimations than that before treatment. The result was confirmed by the Simpson Index (Table 1).

### Beta diversity analysis

**Figure 4:**
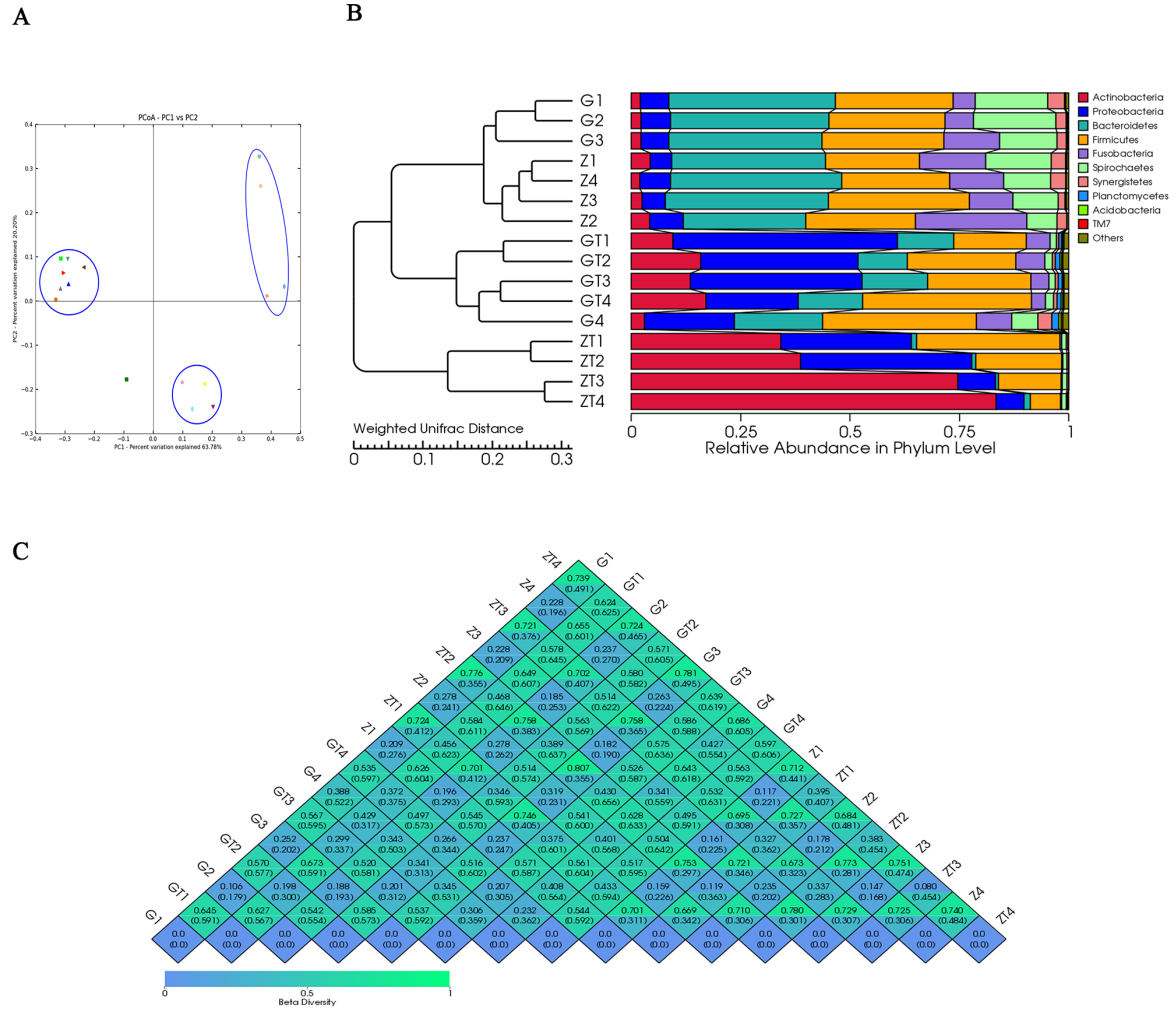
Beta-deversity analysis index **A**. showed Principal coordinate analysis (PCoA) scores plot based in weighted UniFrac metrics. Each symbol represented each sample from patient G and Z before and after treatment. Principal components (PCs) 1 and 2 explained 63.78% and 20.20% of the variance respectively. **B**. showed the phylogenetic tree based on the Weighted UniFrac. The left part is the phylogenetic tree based on the similarity between samples; the right part is the heatmap of relative abundance in phylum level. **C**. showed the β-diversity index heatmap.The numerical values within the squares indicated thecoefficient of difference between each two samples. The green color represented greater difference, and the blue color represented smaller difference.

To further explore the relationship between different bacterial communities pre- and post-treatment, a PCoA analysis was performed using genus-level taxonomic profiles. UniFrac PCoA of 7897 (average 494) OTUs (grouped at 97% sequence identity) showed a clear separation between the samples pre and post-treatment (Figure 4A). Percentage values at the axes indicate contribution of the principal components to the explanation of total variance in the dataset. Figure 4A showed that the percentage of variation explained by PC1 and PC2 were 63.78% and 20.20%. Samples before treatment formed a group apart from the two groups after treatment, which indicated that there was significant difference in the subgingival bacterial composition before and after treatment. The samples of two patients before treatment aggregated in the same group while distracted after treatment, which suggested that the bacterial composition of the two patients was similar before treatment, but exhibited significant difference after treatment. Interestingly, one sample (G4) before treatment was different, and not belonging to any of the three groups. The samples in the group before treatment were well separated from those in the group after treatment based on the Weighted UniFrac distance measured at the OTU level. The phylogenetic tree based on the Weighted UniFrac also revealed the separation between samples pre- and post-treatment, which was in accordance with PCoA result (Figure 4B). Based on the distance between weighted and unweighted UniFrac measured at the OTU level, the Beta diversity index heatmap was generated (Figure 4C). The numerical values within the squares indicated the variation of coefficient between each two samples. 0 represents identity, 1 means totally different. The smaller the discrepancy coefficient was, the more similarity there was between two samples. Within one square, the upper and lower numerical values represented the weighted UniFrac and unweighted UniFrac distances respectively. Figure 4C showed that there was little difference between samples before treatment but more difference in microbial community of the same site after treatment. Moreover, after treatment, the microbial community shared less similarity between two patients (different groups). We also found whenever before or after treatment, the microbial similarity in the same site among the two patients had a less difference than that in one site before and after treatment.

### Variance analysis of species abundance

**Figure 5:**
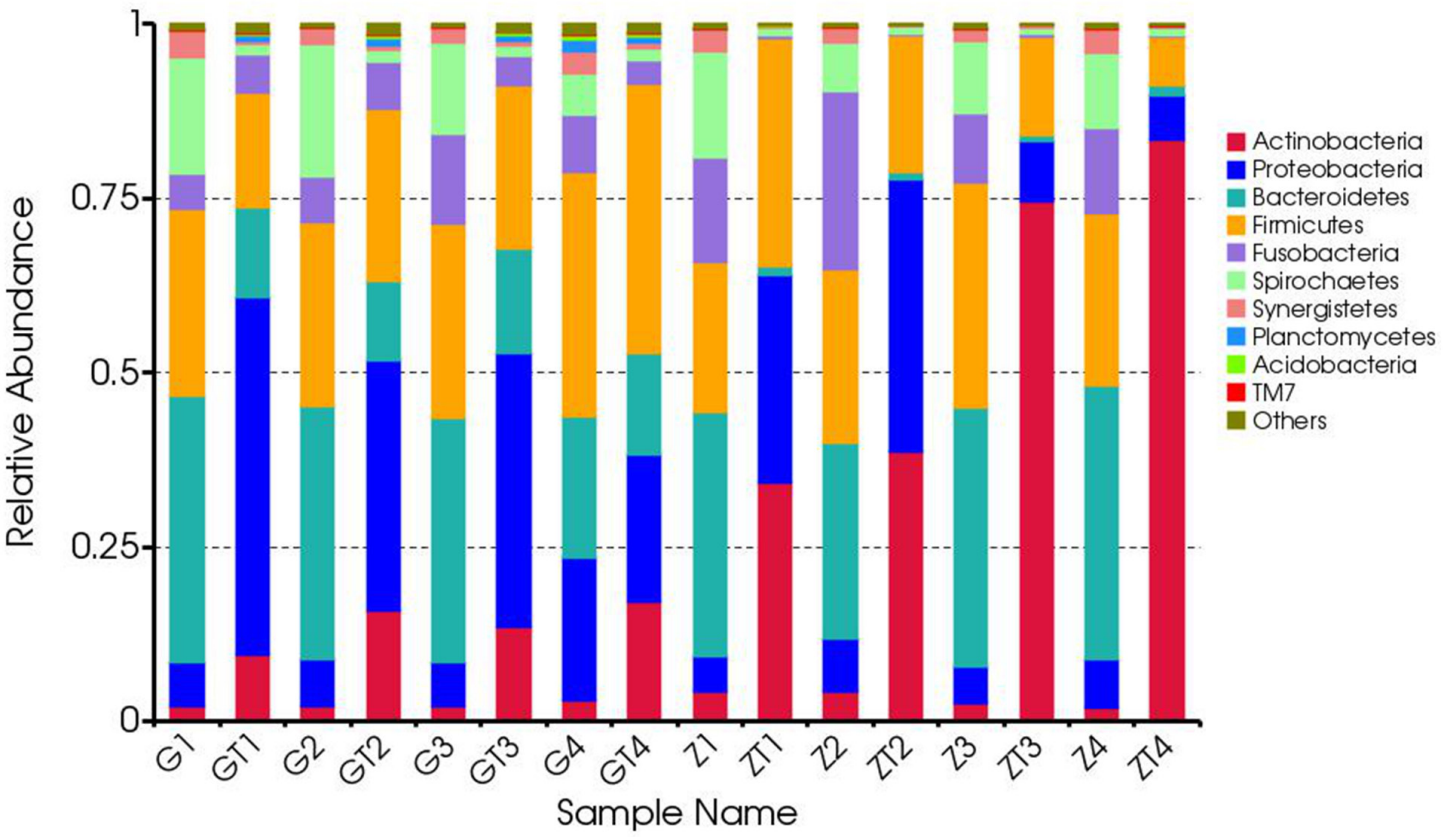
Composition and relative abundance communities based on 16S rDNA sequence in phylum level The distribution of major phyla in the bacterial communities of samples before and after treatment. Sequences that could not be classified into any known group were labeled “other”.

All of the sequences were classified into 34 phyla. The top six most abundant bacteria in phylum level in each sample were shown in Table S4. The relative abundance of each phylum was indicated based on the homogenized OTU numbers (Figure 5). Before treatment, microbial community was dominated by the six most predominant bacteria: *Bacteroidetes* (20.2-39.2%), *Firmicutes* (21.4-35.1%), *Spirochaetes* (6-18.9%), *Proteobacteria* (5-20.5%), *Fusobacteria* (5.1-25.5%) and *Synergistetes* (1.6-3.9%), which was in accordance with previous studies [4, 5]. The remaining genera contributed less than 4.9% in proportion. In contrast, the bacterial community after mechanical debridement exhibited a much different taxonomic composition, in which the most prevalent phyla became: *Actinobacteria* (9.5-83.4%), *Firmicutes* (6.9-38.6%), *Proteobacteria* (6.4-51.3%), *Bacteroidetes* (0.8-15.1%), *Spirochaetes* (1.0-1.9%), *Fusobacteria* (0.2-6.7%) (Table S4). Previous studies revealed a community dominated by the bacterial phyla *Firmicutes*, *Actinobacteria*, *Bacteroidetes*, *Fusobacteria* and *Proteobacteria*in in chronic periodontitis patients [12]. We found these five phyla also dominated the subgingival microbial community of GAgP patients. Moreover, *Spirochaetes* was also one of the predominant bacteria in these two aggressive periodontitis (AgP) patients which was reported previously [4, 5] [13]. Generally speaking, a shift from Gram-negative bacteria to Gram-positive bacteria was observed after treatment in our study. After periodontal therapy, the proportion of *Actinobacteria* and *Proteobacteria* increased dramatically while the proportion of *Bacteroidetes*, *Spirochaetes*, *Fusobacteria* decreased dramatically, which suggested these three anaerobic and Gram-negative bacteria *Bacteroidetes*, *Spirochaetes*, *Fusobacteria* were the putative periodontal pathogens for GAgP. Periodontal pathogens decreased after therapy with the increase of other bacteria, such as *Actinobacteria* and *Proteobacteria*. Actually, we knew that *Porphyromonas gingivalis* (*Bacteroidetes*), *Treponemas denticola* (*Spirochaetes*) and *Fusobacterium nucleatum* (*Fusobacteria*) are the main periodontal pathogens [9]. The most interesting phenomenon appeared when we analyzed the two patients separately. Before treatment, the six most predominant bacterial compositions of the two patients in phylum level were quite similar, though the distribution of each was slightly different. However, after treatment, the bacterial composition of the two patients exhibited a huge difference. For patients Z, *Actinobacteria* became the most dominant bacteria, especially in sites ZT3 and ZT4 (*Actinobacteria* accounts for 75 and 83%) which suggested that the microbial transition after periodontal therapy might vary among different patients.

**Figure 6:**
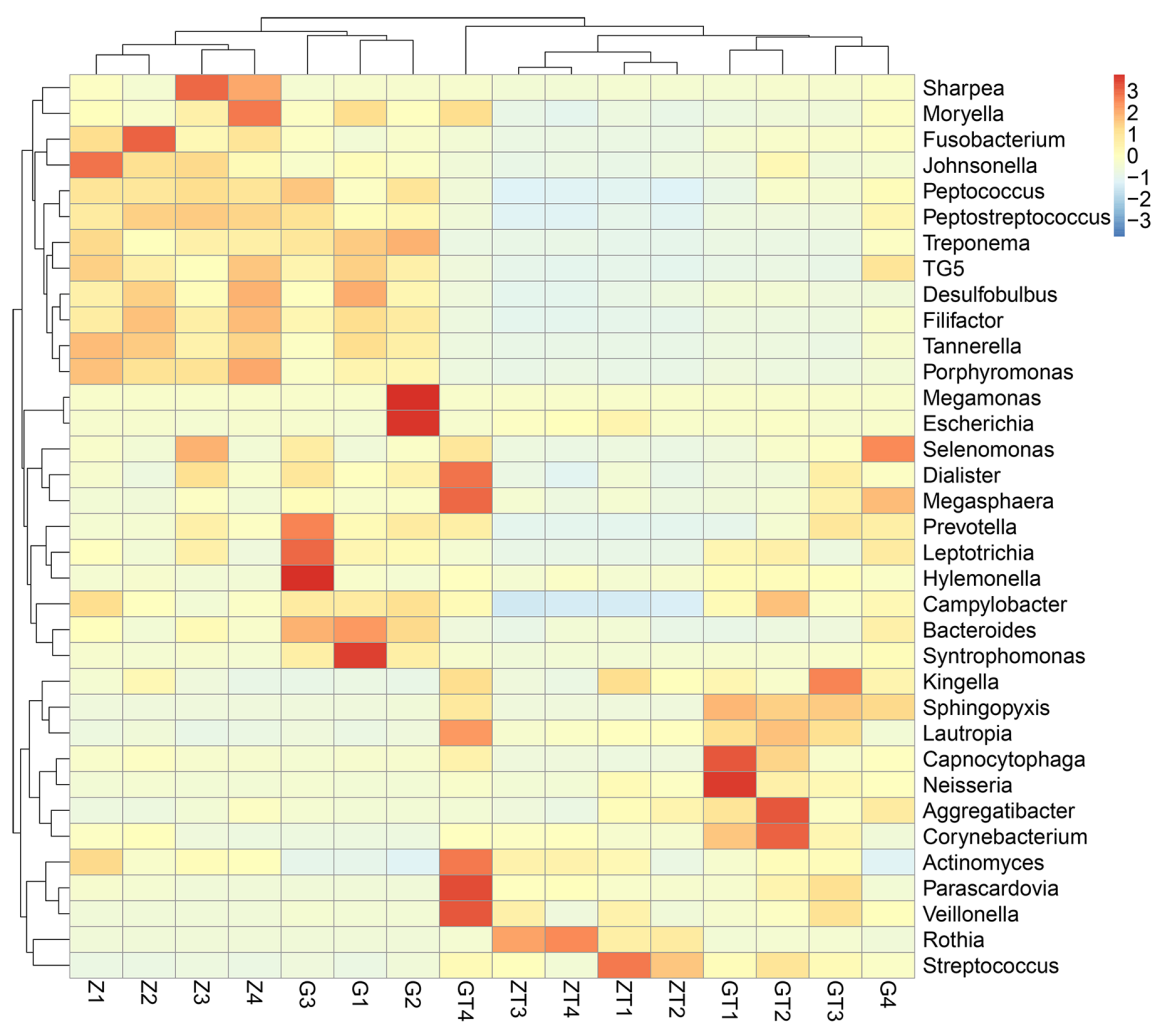
Relative abundance of the top 35 most prevalent genera in subgingival plaque samples before and after intervention Only genera with more than 1% abundance were included. Each Bar displayed the normalized relative abundance, colors reflected relative abundance from low (blue) to high (red).

Based on the species abundance of each sample, we picked the top 35 species in genera level to generate a heatmap (Figure 6). The top 35 most abundant genera represented 57.2-91.35 % of the bacteria in each sample. Different colors represented different levels of relative abundance. As shown in Figure 6, before treatment, *Sharpea*, *Moryella*, *Fusobacterium*, *Johnsonella*, *Peptococcus*, *Peptostreptococcus*, *Treponema*, *TG5*, *Desulfobulbus*, *Filifactor*, *Tannerella*, *Porphyromonas*, *Megamonas*, *Escherichia*, *Selenomonas*, *Dialister*, *Megasphaera*, *Prevotella*, *Leptotrichia*, *Hylemonella*, *Campylobacter*, *Bacteroides*, *Syntrophomonas* had a higher level of abundance. Then, after treatment, *Kingella*, *Sphingopyxis*, *Lautropia*, *Capnocytophaga*, *Neisseria*, *Aggregatibacter*, *Corynebacterium*, *Actinomyces*, *Parascardovia*, *Veillonella*, *Rothia* and *Streptococcus* had a higher level of abundance. We picked the most abundant phylum in each sample and annotated its belonging in phylum level (Table S5).

## DISCUSSION

Many potential pathogens are associated with periodontitis, while traditional culture-dependent methods can not detect all oral microbiomes since most of them have a very little trace or are uncultivable. Metagenosome sequencing based on culture-independent methods can facilitate the study process of microbiome and their functions in the development of periodontitis. As a polymicrobial disease, there are many co-infecting species interacting with each other. The present study was designated to characterize the composition transition of subgingival microbiomes associated with GAgP patients pre- and post-treatment. While only two subjects were involved (with each subject contributing one pooled sample from four periodontal sites pre- and post-treatment), the data was informative and provided a guideline for future studies. We have achieved sufficient sequencing depth to account for any bacterial species that constituted 0.02% or greater of the subgingival microbiota. Given that the sample size for each patient was small, we mainly focused on the comparison of subgingival plaque composition before and after non-surgical periodontal treatment, which might throw light on the study of the interaction between different oral microbiomes associated with periodontitis.

Many studies have identified that *Aggregatibacter. actinomycetemcomitans*, *Porphyromonas. gingivalis*, *Tannerella. forsythia* and *Treponema. denticola* were the predominant pathogens of GAgP [4, 5]. In our study, we found that *Bacteroidetes*, *Firmicutes*, *Spirochaetes*, *Proteobacteria*, *Fusobacteria* and *Synergistetes* were the most 6 relatively abundant phylum before treatment. Studied have characterized that *Aggregatibacter. actinomycetemcomitans* belongs to *Proteobacteria*, *Porphyromonas. gingivalis* and *Tannerella. forsythia* belong to *Bacteroidetes*, and *Treponema. denticola* belongs to *Spirochaetes* in phylum level. Our study was consistent with all of these previous findings. Among these phyla, *Spirochaetes* also has been reported as a dominant bacterium in dental calculus [14]. Since calculus was a predisposing local factor, *Spirochaetes* in dental calculus might be a potential factor leading periodontitis. After treatment, the proportion of *Actinobacteria* and *Proteobacteria* increased dramatically while the proportion of *Bacteroidetes*, *Spirochaetes* and *Fusobacteria* decreased greatly. This phenomenon demonstrated that most of the main pathogens had been removed. We also found that the distribution of microbiomes after treatment between the two patients was different, which suggested that the commensal or normal microbiomes were diverse in different individuals. We suspected that change of physicochemical environments and their influence to each other might alter the distribution of the microbioms since their physicochemical characteristics were very different.

In this study, the subgingival microbiota was high in species richness but dominated by a few species or phylotypes, with significant shifts of microbiota that occurred after treatment. Also, the composition of the subgingival microbiota was highly individualized. The observed species richness in the subgingival microbiota was in general agreement with other studies [2, 4, 5].

Pre- and post-treatment samples showed significant quantitative and distributive species changes. Quantitatively, many known pathogenic species went from easily detectable to zero counts while the opposite occurred for many known health-associated bacteria. In most cases, the abundance of these known pathogens decreased dramatically, with some species, such as *Fusobacterium*, *Treponema*, *Tannerella*, *Porphyromonas*, *Prevotella*, *Campylobacter* and *Bacteroides*. Conversely, known commensal such as *Actinomyces*, *Veillonella* and *Rothia* increased after treatment. Two suspected pathogens, *Aggregatibacter* and *Capnocytophaga* increased after treatment. The assignments of bacterial species to either pathogen or commensal have not always been consistent in the literature. As the clinical parameters improved after treatment, perhaps these species may be considered more beneficial than pathogenic. Some opportunistic pathogen increased after treatment, which can not cause diseases in normal conditions.

When comparing pre- and post-treatment samples, we observed a shift in the composition of the oral microbiota, supporting the well characterized transition from a Gram-negative dominated community in pre-treatment samples, to a Gram-positive dominated community in post-treatment samples. Not surprisingly, Gram-negative genera *Fusobacterium*, *Treponema*, *Tannerella*, *Porphyromonas*, *Prevotella*, *Campylobacter* and *Bacteroides* were significantly enriched in samples before treatment. Among these, three genera, *Treponema*, *Tannerella*, *Porphyromonas*, were of particular interest, as they include the species *Porphyromonas gignivalis*, *Treponema denticola* and *Tannerella forsythia*, proposed to form the pathogenic “red complexes” consortium in periodontitis [15]. Furthermore, the genus *Prevotella* also includes several known periodontal associated pathogens (*Preveotell nigrescens*, *Preveotella intermedia* and *Prevotella melaninogenica*) [16]. For post-treatment samples, Gram-negative genera *Fusobacterium*, *Treponema*, *Tannerella*, *Porphyromonas*, *Prevotella*, *Campylobacter* and *Bacteroides* were remarkably suppressed. Furthermore, a set of genera *Kingella*, *Sphingopyxis*, *Lautropia*, *Capnocytophaga*, *Neisseria*, *Aggregatibacter*, *Corynebacterium*, *Actinomyces*, *Parascardovia*, *Veillonella*, *Rothia* and *Streptococcus* increased abundance in the samples after treatment. Of them, *Streptococcus*, *Actinomyces* and *Rothia* were known as early colonizer species [17]. Surprisingly, both *Aggregatibacter* and *Capnocytophaga* were found to be more abundant after treatment at least in one sample, and one species of *Aggregatibacter* genus was *Aggregatibacter actinomycetemcomitans*, long been considered as a major etiologic agent of GAgP [18]. Since taxonomic resolution down to species-level was impossible to visualize, we were not able to determine if the sequencing reads counting for the genus *Aggregatibacter* is *Aggregatibacter. actinomycetemcomitans*, *Aggregatibacter. aphrophilus* or *Aggregatibacter. segnis*. *Aggregatibacte*r contains the aforementioned three species which was previously implicated as the periodontal pathogen [19]. This phenomenon needs further investigation.

The findings in the present study might provide a novel framework to understand the pathogens and their mechanism of the GAgP from the gene level. However, the size of the samples was limited, more samples were needed to study the interaction between pathogens more deeply and find the functional genes of oral microbiomes.

## MATERIALS AND METHODS

### Study subjects

The study was approved by the Medical Ethical Committee of School of Stomatology, Shandong University (Protocol Number: 201302070). Two subjects (designated as G and Z) with GAgP were recruited from Department of Periodontology, School of Stomatology, Shandong University. Both patients signed an informed consent prior to their enrollment in the study. A diagnosis of GAgP was determined based on the American Academy of Periodontology of Periodontal parameters [8]. Both of the subjects are non-smoking females (27 and 29 years old), who were free of systemic diseases and had not taken antibiotics within the past year for any reason, nor had they received any non-surgical or surgical periodontal therapy previously.

### Clinical examination and treatment

An experienced dentist measured clinical periodontal parameters, delivered treatment and obtained subgingival plaques. Clinical parameters including PD and BOP on six sites per tooth and all teeth were recorded at baseline and one month after therapy. Both patients received full mouth scaling and root planning.

### Microbial sampling

Subgingival plaque samples from mesio-buccal site of the first molar were obtained at baseline and at the four-week follow up so that a total of four samples before treatment and four samples after treatment for each patient were analyzed. Samples were taken from mesio-buccal site of four first molars with initial probing depths ≥ 5mm. Sampling sites were isolated with cotton rolls after all supragingival plaque and calculus were removed using sterile Gracey curettes and placed in 0.5ml PBS. After centrifuged at 3000g for 5min, the supernatants were frozen at -80°Cfor further processing.

### Extraction of genome DNA

Total genome DNA from samples was extracted using CTAB method. DNA concentration and purity was monitored on 2% agarose gel. According to the concentration, DNA was diluted to 1ng/μl using sterile water.

### Bacterial 16s rDNA gene amplification and Illumina Sequencing

We picked the fragments containing V4 16s rDNA hypervariable region for gene amplification [20, 21]. The sequencing method was described by Caporaso et al [22]. The primer set selected for amplifying the V4 16s rDNA hypervariable region was 515f/806r. It exhibited few biases and yielded accurate phylogenetic and taxonomic information. The reverse primer contained a 6-bp barcode which was unique to each sample. All PCR reactions were carried out in 30μl reaction system with 15μl of Phusion^®^ High-Fidelity PCR Master Mix (New England Biolabs), 0.2μmol l^-1^of forward and reverse primers, and about 10ng template DNA. Thermal cycling consisted of initial denaturation at 98°C for 1min, followed by 30 cycles of denaturation at 98°C for 10s, annealing at 50°C for 30s, and elongation at 72°C for 60s and finally 72°C for 5min. The quantification and qualification of PCR products were carried on by operating electrophoresis on 2% agarose gel. PCR products were mixed in equidensity ratios. Then, mixed PCR products were purified with V4 hypervariable region was picked for PCR amplification of subgingival samples GeneJET Gel Extraction Kit (Thermo Scientific). Finally, the library was sequenced by Illumina MiSeq platform and 250bp/300bp paired-end reads were generated.

### Data analysis

Paired-end reads from the original DNA fragments were merged by FLASH V1.2.7, http://ccb.jhu.edu/software/FLASH/ [23] -a very fast and accurate analysis tool which was designed to merge paired-end reads when there were overlaps between two reads (reads 1 and reads 2). After strict quality-filtering [24], paired-end reads were assigned to each sample according to the unique barcodes. Sequences were analyzed using QIIME software package V1.7.0, http://qiime.org/scripts/split_libraries_fastq.html First, reads were filtered by QIIME quality filters. The process involved four sections:1) min_per_read_length: minimum number of consecutive high-quality base calls to retain read (as percentage of total read length). 2) max_bad_run_length: maximum number of consecutive low quality base calls allowed before truncating a read. 3) sequence_max_n: maximum number of ambiguous (N) characters allowed in a sequence. 4) phred_quality_score: last quality score considered low quality [25]. Then we used UPARSE pepline V7.0, 1001, http://drive5.com/uparse/ [26] to pick operational taxonomic units (OTUs) by making OTU table. Sequences with ≥97% similarity were assigned to the same OTUs. We picked a representative sequences for each OTU and used Ribosomal Database Project (RDP) classifier V2.2, http://sourceforge.net/projects/rdp-classifier [27] and Greengenes database http://greengenes.lbl.gov/cgi-bin/nph-index.cgi [28] to annotate taxonomic information for each representative sequence. We used MUSCLE V3.8.31, http://www.drive5.com/muscle/ [29, 30] software to get the phylogenetic relationship based on the respective OTUs.

In order to compute Alpha diversity, we rarified the OTU table and calculate three metrics by QIIME: Shannon index estimates the diversity and evenness; Observed Species estimates the amount of unique OTUs found in each sample, and Chao1 estimates the richness. Rarefaction curves were generated based on these three metrics. At the same time, we calculated the Simpson's Index to visualize evenness.

QIIME calculates both weighted and unweighted unifrac [31–33], which are phylogenetic measures of beta diversity. We used weighted unifrac for Principal Coordinate Analysis (PCoA) and Unweighted Pair Group Method with Arithmetic mean (UPGMA). PCoA helps to get principal coordinates and visualize them from complex, multidimensional data. It takes a transformation from a distance matrix to a new set of orthogonal axes. By which the maximum variation factor is demonstrated by first principal coordinate, and the second maximum one by the second principal coordinate, and so on. UPGMA is a type of hierarchical clustering method using average linkage and can be used to interpret the distance matrix. A schematic overview of the experimental work-flow and applied bioinformatic procedure was given in Figure S1.

## SUPPLEMENTARY TABLES AND FIGURES


