# Supplementary material for: The microbial community shifts of subgingival plaque in patients with generalized aggressive periodontitis following non-surgical periodontal therapy: a pilot study
Source: Oncotarget. 2016 Oct 8;8(6):10609–19. doi: 10.18632/oncotarget.12532 (PMC5354685; doi:10.18632/oncotarget.12532)
Supplement: Supplementary file 1 [file oncotarget-08-10609-s001.pdf]

# The microbial community shifts of subgingival plaque in patients with generalized aggressive periodontitis following non-surgical periodontal therapy: a pilot study

## Supplementary Material

**Table S1 Demographic patient data and clinical parameters at baseline pre- and post-treatment.** Two patients were females, non-smokers with generalized aggressive periodontits GAgP.For every tooth clinical parameters (PD, BOP) were determined from six sites.

| Patient | G | Z |
|---|---|---|
| Age(years) | 29 | 27 |
| Num of teeth | 27 | 30 |
| Percent of sites with PD≤3mm （pre/post intervention) | 15.43/93.21 | 13.33/55.56 |
| Percent of sites with PD 4~6mm （pre/post intervention) | 54.94/6.79 | 52.78/40.56 |
| Percent of sites with PD≥7mm （pre/post intervention) | 29.63/0 | 33.89/3.89 |
| Percent of sites with BOP（pre/post intervention) | 100/0 | 100/9 |

**Table S2 Filtration and quality evaluation of original data.** Raw PE-reads meant the original paired-end reads after amplification. Combined meaned the number of tags after merging paired-end reads.Qualified meant the number of tags after filtering. Nochime meant number of tags after filtering chimera, which were used for the subsequent analysis (clean tags). Base meant the number of base of clean number. AvgLen meant the average length of the clean tags. Q20 meant the percentage of base

whose error rate of sequencing less than 1% and 0.1% respectively. GC (%) meant the percentage of GC base in clean tags. Effective (%) meant the rate of tags in clean tags and the paired-end reads in raw data.

| Sample Name | Raw PE-reads | Combined | Qualified | Nochime | Base(nt) | AvgLen(nt) | Q20 | Q30 | GC% | Effective% |
|---|---|---|---|---|---|---|---|---|---|---|
| G1 | 63,256 | 62,606 | 62,161 | 61,062 | 15,433,413 | 253 | 98.18 | 94.9 | 53.11 | 96.53 |
| G2 | 40,214 | 39,832 | 39,588 | 38,841 | 9,819,301 | 253 | 98.24 | 95.04 | 52.49 | 96.59 |
| G3 | 31,160 | 30,879 | 30,687 | 30,046 | 7,593,432 | 253 | 98.27 | 95.03 | 52.39 | 96.42 |
| G4 | 47,249 | 46,734 | 46,440 | 45,307 | 11,446,912 | 253 | 98.27 | 95.06 | 53.18 | 95.89 |
| GT1 | 95,594 | 94,588 | 94,049 | 91,527 | 23,146,405 | 253 | 98.38 | 95.34 | 52.66 | 95.75 |
| GT2 | 100,089 | 98,879 | 98,214 | 95,122 | 24,050,849 | 253 | 98.19 | 94.9 | 53.21 | 95.04 |
| GT3 | 74,434 | 73,665 | 73,201 | 70,914 | 17,926,909 | 253 | 98.35 | 95.24 | 53.66 | 95.27 |
| GT4 | 73,753 | 72,993 | 72,522 | 70,684 | 17,860,257 | 253 | 98.23 | 94.99 | 54.44 | 95.84 |
| Z1 | 61,163 | 60,655 | 60,324 | 58,797 | 14,857,091 | 253 | 98.32 | 95.2 | 51.79 | 96.13 |
| Z2 | 47,946 | 47,453 | 47,177 | 46,158 | 11,657,141 | 253 | 98.3 | 95.16 | 51.08 | 96.27 |
| Z3 | 67,923 | 67,230 | 66,804 | 65,444 | 16,532,347 | 253 | 98.16 | 94.83 | 52.38 | 96.35 |
| Z4 | 44,534 | 44,095 | 43,821 | 42,540 | 10,747,502 | 253 | 98.18 | 94.93 | 52 | 95.52 |
| ZT1 | 12,564 | 12,443 | 12,370 | 12,171 | 3,076,014 | 253 | 98.25 | 95.03 | 54.12 | 96.87 |
| ZT2 | 76,962 | 76,333 | 75,930 | 74,646 | 18,872,811 | 253 | 98.2 | 94.87 | 54.49 | 96.99 |
| ZT3 | 77,586 | 76,915 | 76,438 | 75,552 | 19,106,478 | 253 | 98.1 | 94.65 | 55.71 | 97.38 |
| ZT4 | 37,845 | 37,509 | 37,238 | 36,815 | 9,313,307 | 253 | 98.04 | 94.47 | 56.04 | 97.28 |

**Table S3 The percentage of shared OTUs among groups.** The G and Z represented the samples from the patient G and Z before treatment; the GT and ZT represented the samples from the patient G and Z after treatment. The shared OTUs in sample of G1-G3 and Z1-Z4 decreased after treatment. While, the shared OTUs in sample of G4 increased after treatment.

| **Group percentage number** | **1** | **2** | **3** | **4** |
|---|---|---|---|---|
| G | 82.3 | 79.1 | 80.8 | 42.6 |
| GT | 62.5 | 64.7 | 60.9 | 63.0 |
| Decline | ↓ | ↓ | ↓ | ↑ |
| Z | 76.0 | 76.6 | 76.3 | 79.6 |
| ZT | 50.8 | 67.2 | 63.4 | 42.5 |
| Decline | ↓ | ↓ | ↓ | ↓ |

**Table S4 The top 6 most abundant bacteria in phylum level in each sample.** G and Z represented the samples from the patient G and Z before treatment; GT and ZT represented the samples from the patient G and Z after treatment.

| **（G1\G2\G3\G4）** | **percentage** | **（GT1\GT2\GT3\GT4）** | **percentage** |
|---|---|---|---|
| *Bacteroidetes* | 0.2017-0.3810 | *Bacteroidetes* | 0.1121-0.1508 |
| *Firmicutes* | 0.2646-0.3512 | *Firmicutes* | 0.1654-0.3858 |
| *Spirochaetes* | 0.0597-0.1894 | *Spirochaetes* | 0.0150-0.0186 |
| *Proteobacteria* | 0.0638-0.2047 | *Proteobacteria* | 0.2110-0.5134 |
| *Fusobacteria* | 0.0507-0.1279 | *Fusobacteria* | 0.0325-0.0669 |
| *Synergistetes* | 0.0213-0.0391 | *Actinobacteria* | 0.0951-0.1708 |
| （Z1\Z2\Z3\Z4） | percentage | （ZT1\ZT2\ZT3\ZT4） | percentage |
| *Bacteroidetes* | 0.2808-0.3918 | *Bacteroidetes* | 0.0076-0.0139 |
| *Firmicutes* | 0.2140-0.3223 | *Firmicutes* | 0.0695-0.3277 |
| *Fusobacteria* | 0.0996-0.2556 | *Fusobacteria* | 0.0023-0.0040 |
| *Spirochaetes* | 0.0688-0.1504 | *Spirochaetes* | 0.0095-0.0109 |
| *Proteobacteria* | 0.0497-0.0763 | *Proteobacteria* | 0.0642-0.3914 |
| *Synergistetes* | 0.0156-0.0342 | *Actinobacteria* | 0.3415-0.8339 |

**Table S5 The shift of the most dominant microbial in genus level pre- and pot-treatment and their belonging in phylum level.** G and Z represented the samples from patients G and Z before treatment; GT and ZT represented the samples from patients G and Z after treatment.

| | **Before treatment** | **After treatment** | | **Before treatment** | **After treatment** |
|---|---|---|---|---|---|
| **Z1-ZT1** | *Johnsonella*（*Actinobacteria*） | *Streptococcus*（*Actinobacteria*） | G1-GT1 | *Syntrophomonas*（*Actinobacteria*） | *Neisseria*（*Proteobacteria*） |
| **Z2-ZT2** | *Fusobacterium*（*Fusobacteria*） | *Streptococcus*（*Actinobacteria*） | G2-GT2 | *Megamonas*（*Actinobacteria*） | *Aggregatibacter*（*Proteobacteria*） |
| **Z3-ZT3** | *Sharpea*（*Actinobacteria*） | *Rothia*（*Actinobacteria*） | G3-GT3 | *Hylemonella*（*Proteobacteria*） | *Kingella*（*Proteobacteria*） |
| **Z4-ZT4** | *Moryella*（*Actinobacteria*） | *Rothia*（*Actinobacteria*) | G4-GT4 | *Selenomonas*（*Actinobacteria*） | *Parascardovia*（*Actinobacteria*） |

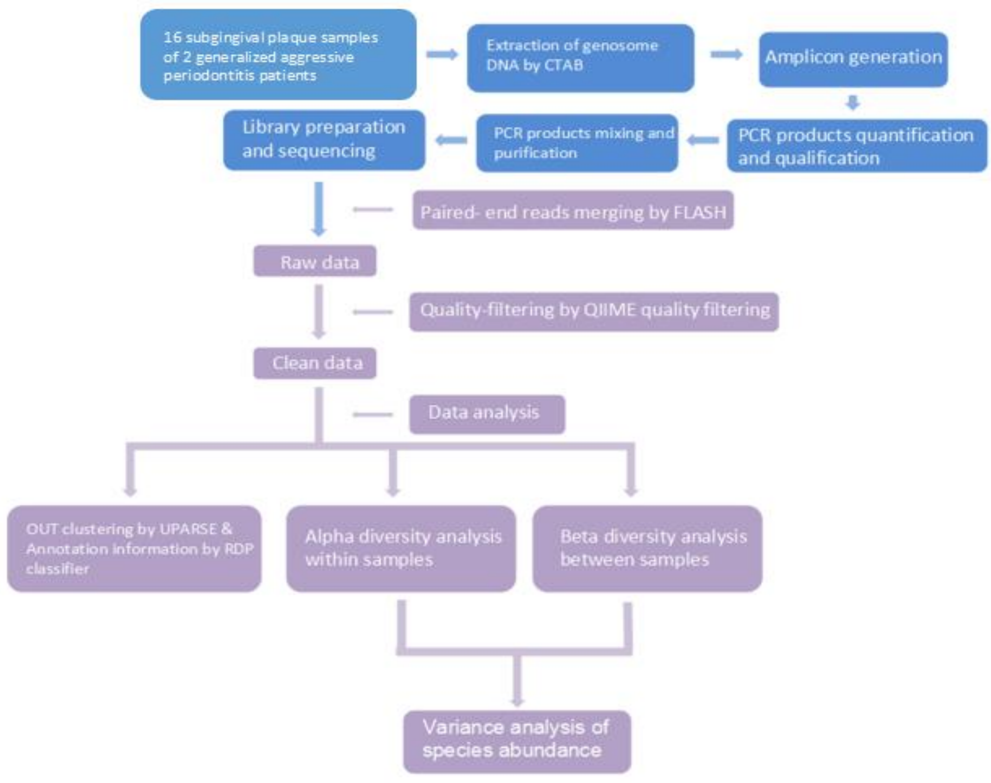


**Figure S1. The schematic overview of the experimental work-flow and applied bioinformatic procedure.** The blue color meaned the part of sequencing, and the purple color meaned the part of bioinformatic analysis.

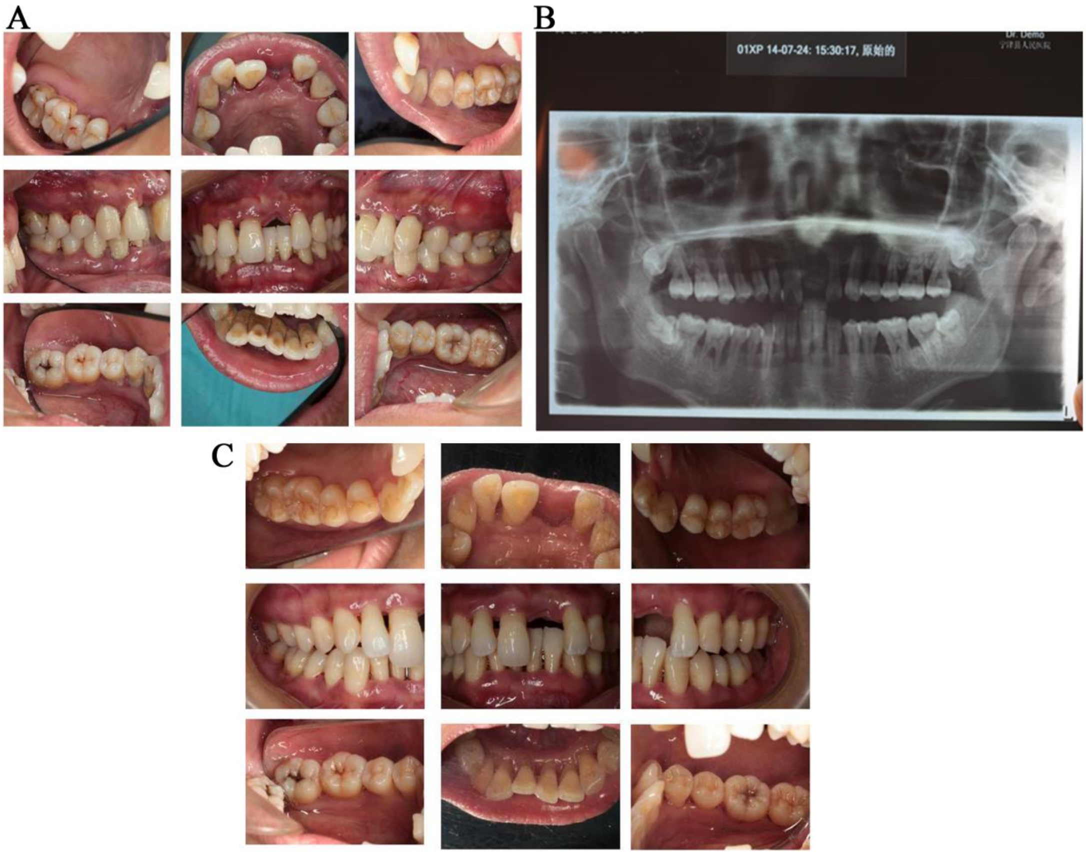


**Figure S2. The intraoral photos and panoramic radiographs of patient G.** (A) showed the intraoral photo before treatment. (B) showed the panoramic radiograph before treatment. (C) showed the intraoral photo after treatment.

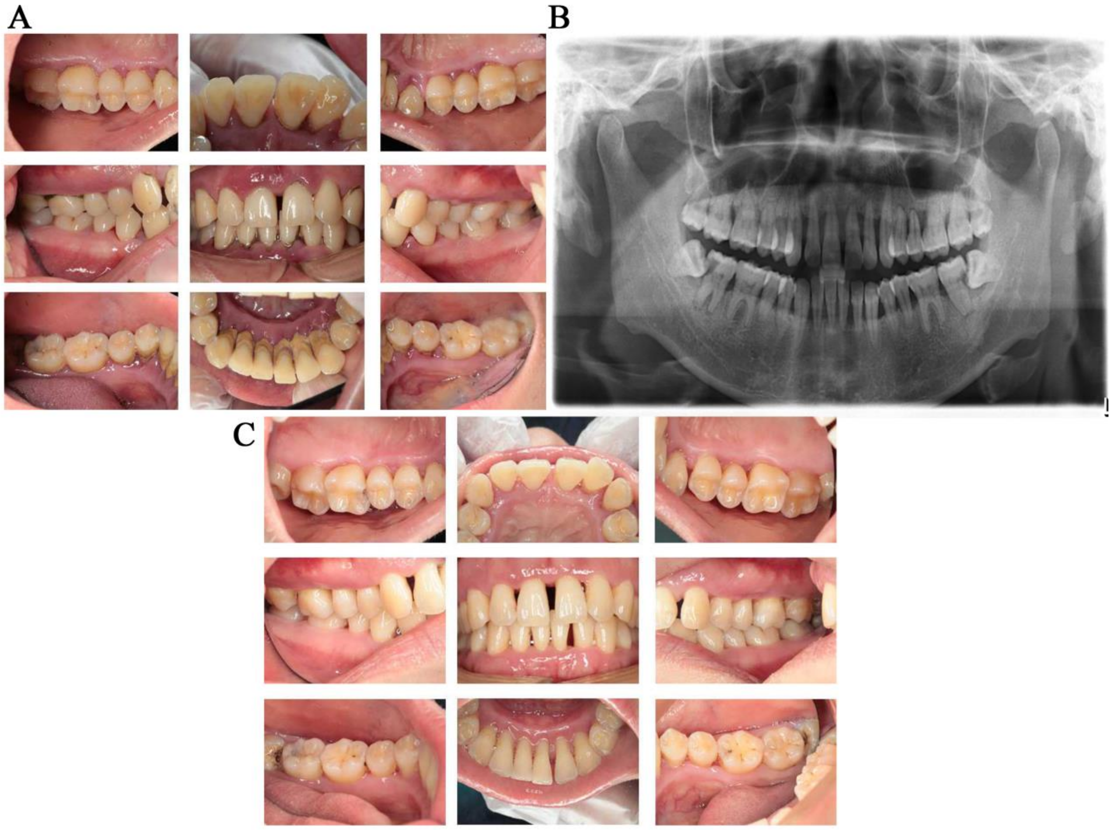


**Figure S3. The intraoral photos and panoramic radiographs of patient Z.** (A) showed the intraoral photo before treatment. (B) showed the panoramic radiograph before treatment. (C) showed the intraoral photo after treatment.
